# Oxidative Stress and Hypoxia Contribute to Alzheimer's Disease Pathogenesis: Two Sides of the Same Coin

**DOI:** 10.1100/tsw.2009.93

**Published:** 2009-08-11

**Authors:** Michela Guglielmotto, Elena Tamagno, Oliviero Danni

**Affiliations:** Department of Experimental Medicine and Oncology, University of Torino, Italy

**Keywords:** Alzheimer’s disease, hypoxia, oxidative stress, BACE1

## Abstract

While it is well established that stroke and cerebral hypoperfusion are risk factors for Alzheimer's disease (AD), the molecular link between ischemia/hypoxia and amyloid precursor protein (APP) processing has only been recently established. Here we review the role of the release of reactive oxygen species (ROS) by the mitochondrial electron chain in response to hypoxia, providing evidence that hypoxia fosters the amyloidogenic APP processing through a biphasic mechanism that up-regulates β-secretase activity, which involves an early release of ROS and an activation of HIF-1α.

## INTRODUCTION

Alzheimer's disease (AD) is classified into two forms: sporadic late-onset AD, which is correlated to aging, and rare familial early-onset AD (FAD), caused by gene mutations. The pathology of AD is defined by the presence of extracellular plaques composed by amyloid-β (Aβ) fibrils, intracellular neurofibrillary tangles (NFTs) composed of hyperphosphorylated tau protein, and synaptic loss.

Central to the disease is the altered proteolytic processing of the Aβ precursor protein (APP), resulting in overproduction and aggregation of neurotoxic forms of Aβ. APP is an integral membrane protein with a single, membrane-spanning domain; a large, extracellular N-terminus; and a shorter, cytoplasmic C-terminus. The amyloidogenic processing of APP involves two sequential cleavages operated by the β- and γ-secretases at the N- and C-termini of Aβ.

The β-secretase (BACE1) cleaves APP at the beginning of the Aβ sequence, generating an extracellular soluble fragment, called sβAPP, and an intracellular C-terminal end, termed C99. C99 is further cleaved within the membrane by the γ-secretase. The γ-cleavage produces Aβ fragments of different length, predominantly Aβ 40 and Aβ 42.

The role of Aβ in the pathogenesis of AD is supported by two major clues. Aggregates of Aβ are neurotoxic and initiate a series of events, including the hyperphosphorylation of tau, which result in neuronal dysfunction and cell death[1]. APP gene overdosage as well as the gene mutations that cause FAD facilitate the accumulation of Aβ 42, increasing its production and aggregation[2,3,4]. The cause of modified APP processing and Aβ 42 accumulation in sporadic cases of AD is unclear, but is likely to include oxidative stress (OS). Thus, OS results from several cellular insults, such as hyperglycemia, glutamate, prion proteins, and calcium homeostasis perturbation[5,6,7,8,9], and our study strengthens the hypothesis that OS is a basic common pathway of Aβ accumulation, as effected by different AD risk factors .

## AB AND OS IN AD/AGING BRAIN

OS increases with age through variations in reactive oxygen species (ROS) generation, ROS elimination, or both[10]. The free radical hypothesis of aging implies that accumulation of ROS results in damage of the major cell components: nucleus, mitochondrial DNA, membranes, and cytoplasmic proteins[11]. The imbalance between the generation of free radicals and antioxidants has been claimed as a cause of AD. The brain is particularly vulnerable to OS because of its high utilization of oxygen, increased levels of polyunsaturated fatty acids, and relatively low levels of antioxidants[12,13].

OS and Aβ are linked to each other since Aβ aggregation induces OS *in vivo* and *in vitro*[14,15,16], and oxidants increase the production of Aβ[17,18,19,20,21]. Transition metals, Cu(II), Zn(II), and Fe(III), favor the neurotoxicity of Aβ through their reduction, which produces hydrogen peroxide ($H_2O_2$)[22]. Using density functional theory calculations, it has been shown that the Aβ residue Tyr-10 is a pivotal residue to drive the catalytic production of $H_2O_2$ by Aβ peptides in the presence of Cu(II). The phenoxy radical of Tyr-10 produced by the reaction with ROS causes neurotoxicity and results in the formation of dityrosine, which accelerates the aggregation of Aβ peptides[23]. Another crucial Aβ residue is Met-35; the substitution of Met-35 with cysteine resulted in no protein oxidation in *C. elegans* model[24]. Moreover, the inhibition of cytochrome c oxidase by Aβ 42 involves the formation of a redox active methionine radical[25]. Lipid peroxidation induced by Aβ peptides impairs the function of ATPases, glucose and glutamate transporters, and also GTP-binding proteins as the result of covalent modification of the proteins by the aldehydic end products, such as 4-hydroxynonenal (HNE)[15,26].

Moreover, Aβ peptides favor $Ca^{2+}$ influx into neurons by inducing membrane-associated OS, rendering neurons vulnerable to excitotoxicity and apoptosis[27].

On the other hand, OS may also be the cause of Aβ accumulation. Oxidant agents and oxidative products increase APP expression[28,29], and intracellular and secreted Aβ levels in neuronal and non-neuronal cells[17,18,19,20,21,30]. We and others have shown that the expression and activity of BACE1 is increased by oxidants[21,31,32,33,34]. Moreover, there is a significant correlation of BACE1 activity with oxidative markers in sporadic AD brain tissue[35], in which a significant increase of BACE1 expression has been shown[36,37,38,39].

## HYPOXIA IN AD/AGING BRAIN

It is well known that patients with stroke and cerebral infarction are at risk of AD[9]. Hypoxia is a direct consequence of hypoperfusion, which plays a role in the Aβ accumulation.

Oxygen homeostasis is essential for the development and functioning of an organism. Hypoxia inducible factor (HIF)-1α is a molecule that regulates oxygen homeostasis[40]. HIF-1α is a member of the basic helix-loop-helix transcription factor family, and the basic region of the protein binds specifically to the 5'-RCGTGb hypoxia-responsive element (HRE) in a gene promoter region. HIF-1 contains an oxygen-regulated expression subunit α (HIF-1α) and a constitutively expressed subunit β (HIF-1β) (Arnt). HIF-1α protein, mediated by its oxygen-dependent degradation domain, is rapidly degraded through the ubiquitin-proteosome pathway under normoxic conditions with a half-life of <5 min, but is quite stable under hypoxic conditions. The oxygen-dependent degradation of HIF-1α is a substrate for post-translational modification by prolyl hydroxylases (PHDs), enzymes that hydroxylate HIF-1α. This hydroxylated form may be recognized by the E3 ubiquitin protein ligase, called the von Hippel-Lindau (pVHL) protein, which signals HIF-1α for degradation[40,41,42,43,44] (Fig. 1).

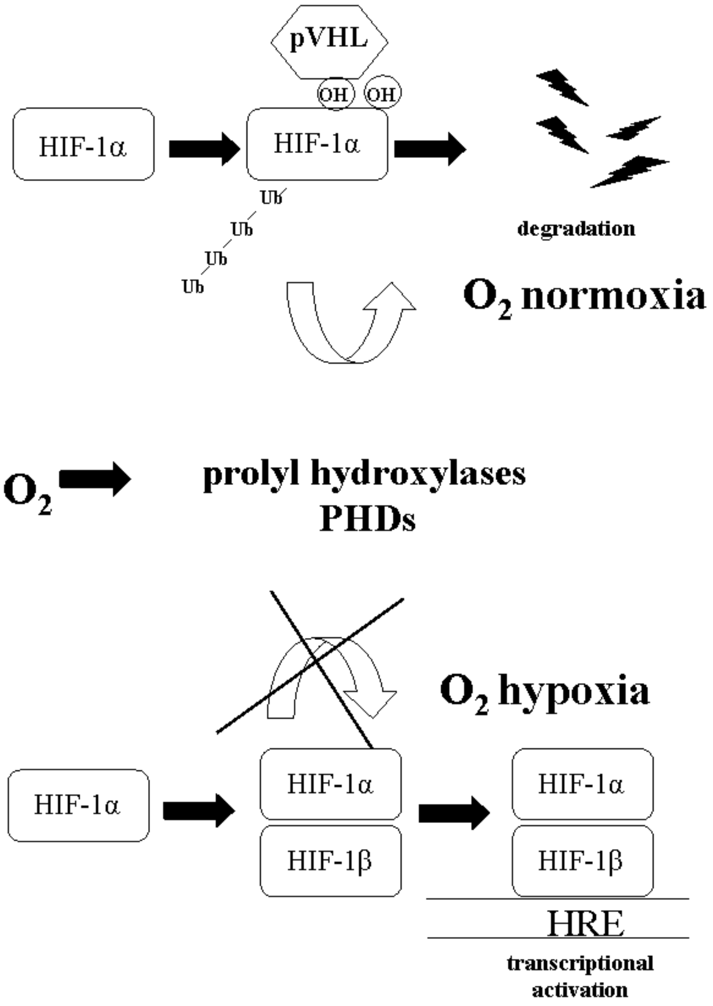

**FIGURE 1**. HIF-1α pathway in normoxic and hypoxic conditions.

Prolonged and severe hypoxia can cause neuronal loss and memory impairment[45]. Recent studies have shown that a history of stroke can increase AD prevalence by approximately twofold in elderly patients[46,47]. The risk is higher when stroke is concomitant with atherosclerotic vascular risk factor[48]. Hypoxia is a direct consequence of hypoperfusion, a common vascular component among the AD risk factors.

Recently, it has been reported that hypoxia can alter APP processing, increasing the activity of the β- and γ-secretases. Sun and collaborators[49] showed that hypoxia significantly up-regulates BACE1 gene expression, resulting in increased β-secretase activity. Moreover, the same authors found that hypoxia increases Aβ deposition and neuritic plaque formation, as well as memory deficit, in Swedish mutant APP transgenic mice. These data provide a molecular mechanistic link of vascular factors with AD.

More recently, sequence analysis and gel shift studies revealed binding of HIF-1α to the BACE1 promoter. It has been shown that overexpression of HIF-1α in neuronal cells increases BACE1 mRNA and protein levels, whereas down-regulation of HIF-1α reduces the levels of BACE1[50]. Hypoxic conditions were also shown to increase the γ-secretase activity.

HIF-1α binds to the anterior pharynx-defective phenotype (APH-1) promoter to up-regulate its expression. The activation of HIF-1α induced by hypoxia increases the expression of APH-1 mRNA and protein, leading to an increased γ-cleavage of APP and Notch[51,52]. APH-1 is a key component of the γ-

secretase complex, together with presenilin, nicastrin (Nct), and presenilin enhancer (PEN) 2, a multimembrane-spanning protein that is required for the correct subcellular transport of Nct to the cell surface[53,54].

Collectively, these data showed that hypoxia increased the β- and γ-secretase activities, which facilitate the abnormal cleavage of APP, resulting in the acceleration of Aβ production and plaque formation both *in vivo* and *in vitro*.

## THE PARADOX OF INCREASED ROS DURING HYPOXIA

Although it is generally accepted that intracellular ROS levels change during hypoxia, the direction in which this change occurs is still hotly debated.

Levels of intracellular ROS paradoxically increase under hypoxia[55,56,57,58,59]. Early theories proposed that NADPH oxidase might be an important ROS-generating cellular oxygen sensor since this multisubunit membrane-bound enzyme is expressed in tissues implicated in systemic hypoxic responses and affects cellular redox status depending on cellular oxygen concentrations[60,61] However, other studies did not support the involvement of NADPH in the hypoxic adaptive response[62]. Chandel et al.[55] suggested that mitochondria are the source of ROS involved in the hypoxic response. The electron transport chain, which is embedded in the inner membrane of mitochondria, consists of five multiprotein complexes. Complexes I and II oxidize the energy-rich molecules NADH and $FADH_2$, respectively, and transfer the resulting electrons to ubiquinol, which carries them to complex III. Complex III, in turn, shuttles the electrons across the inner mitochondrial membrane to cytochrome c, which carries them to complex IV. Complex IV uses the electrons to reduce oxygen to water. Along with carrying electrons, complexes I, II, and III generate ROS[63,64]. It is now accepted that hypoxia increases ROS via the transfer of electrons from ubisemiquinone to molecular oxygen at the Qo site of complex III of the mitochondrial transport chain[65].

The mitochondria-derived ROS are both necessary and sufficient to stabilize and activate HIF-1α.

It has been demonstrated that antioxidants reverse hypoxia-induced HIF-1α activation[66,67]. Recent antitumorigenic effects of antioxidants have been attributed to the inhibition of HIF-1α–dependent events[68]. Moreover, the addition of oxidants, such as $H_2O_2$, induces HIF-1α activity up-regulation in normoxia[69,70].

Mitochondrial ROS regulate HIF-1α stability on hypoxia through different mechanisms. These most likely involve the PHDs, the oxidases involved in the post-translational modification that signals HIF-1α for degradation[71,72]. Recent work showed that mitochondrial inhibitors, such as rotenone and myxothiazol, and the antioxidant MitoQ, maintain PHD activity in hypoxic cells[73]. This mechanism is supported by the fact that genetic removal of the JunD-dependent antioxidant pathway leads to increased HIF-1α activation through decreased activity of the HIF PHDs[74,75]. Under hypoxic conditions, mitochondrial ROS can also activate signaling pathways upstream of HIF-1, such as the extracellular signal-regulated mitogen activated kinase (ERK) and the p38 stress-activated MAPK pathways. ERK2 phosphorylates HIF-1α *in vitro* and *in vivo*, and increases its transcriptional activity[76,77,78].

Regulation of HIF-1α stability and function under hypoxic conditions is also modulated by the phosphatidylinositol 3 kinase (PI3K)-protein kinase B (PKB/Akt) signaling pathway[79]. Several studies have shown that ROS generation can activate this pathway and lead to enhancement of HIF-1α in cancer cells[80,81,82]. PKB/Akt has several targets, such as the forkhead transcription factor 4 (FOXO4), the glycogen synthase kinase 3β (GSK3 β), the oncoprotein human double minute 2 (HDM2), and tuberin (TSC2), a key component of the mTOR (mammalian target of rapamacyn) pathway. Although there is no evidence for direct phosphorylation of HIF-1α by the PKB/Akt pathway, all the above-mentioned downstream effectors of the pathway influence HIF-1α activity (Fig. 2).

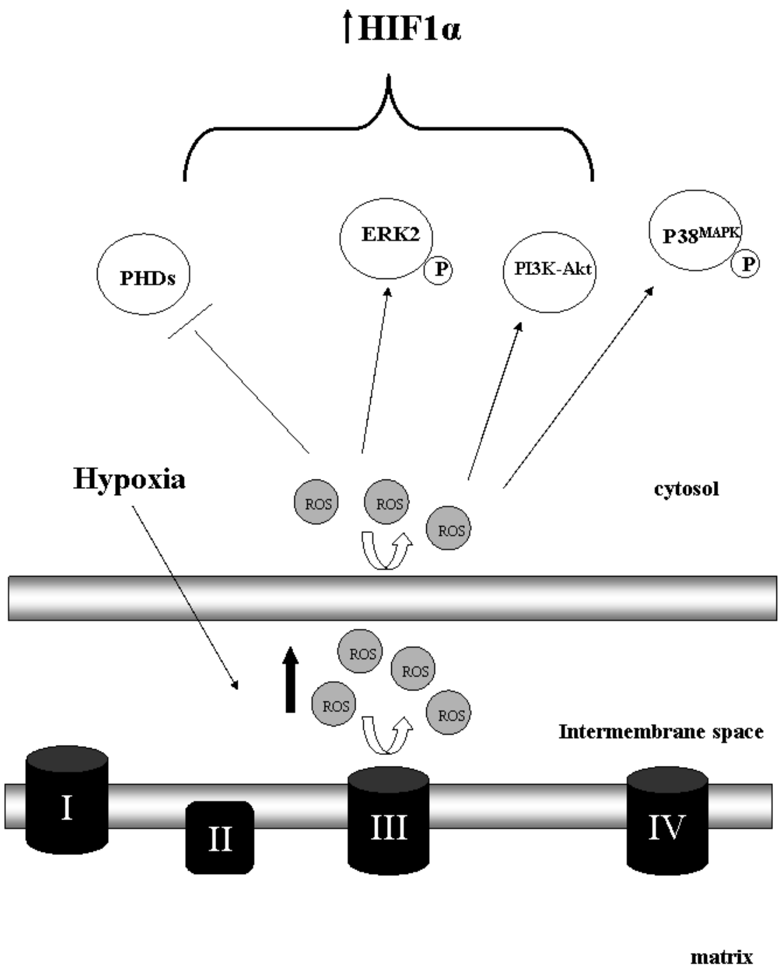

**FIGURE 2**. Schematic outline of the pathways that integrate ROS signaling and HIF-1α pathway in hypoxic condition.

## BACE1 INDUCTION BY HYPOXIA-MEDIATED ROS

Hypoxia increases BACE1 as well as the γ-secretase expression and activity, resulting in Aβ overproduction, as has been shown *in vitro* as well as in AD transgenic mice[49,50,51,52]. HIF-1α binds to the BACE1 promoter and regulates its gene expression[50]. Of note, acute ischemia up-regulates BACE1 activity also through the impaired degradation of BACE1 mediated by the decreased levels of GGA3, a trafficking molecule that delivers BACE1 to the endosomal-lysosomal system[83].

We significantly extended these findings both *in vivo* and *in vitro*, showing that hypoxia up-regulates BACE1 mRNA expression in a biphasic manner, through two distinct mechanisms: (1) an early release of ROS from mitochondria and (2) a late activation of HIF-1α[84].

Our hypothesis is that the early posthypoxic up-regulation of BACE1 depends on the generation of ROS mediated by the sudden interruption of the mitochondrial electron transport chain. The involvement of ROS released by mitochondria was confirmed by complete protection exerted by rotenone and diphenyl-phenylen iodonium, compounds that affect complex I of the mitochondrial electron transport chain[85,86].The later expression of BACE1 is caused by HIF-1α activation.

Thus, contrary to other reports[49,50], in our experimental conditions, we did not detect an evident nuclear translocation of HIF-1α within 12 h of hypoxia. In fact, silencing of HIF-1α (able to block the late up-regulation of BACE1) did not prevent the early increase in BACE1 mRNA and protein levels (Fig. 3).

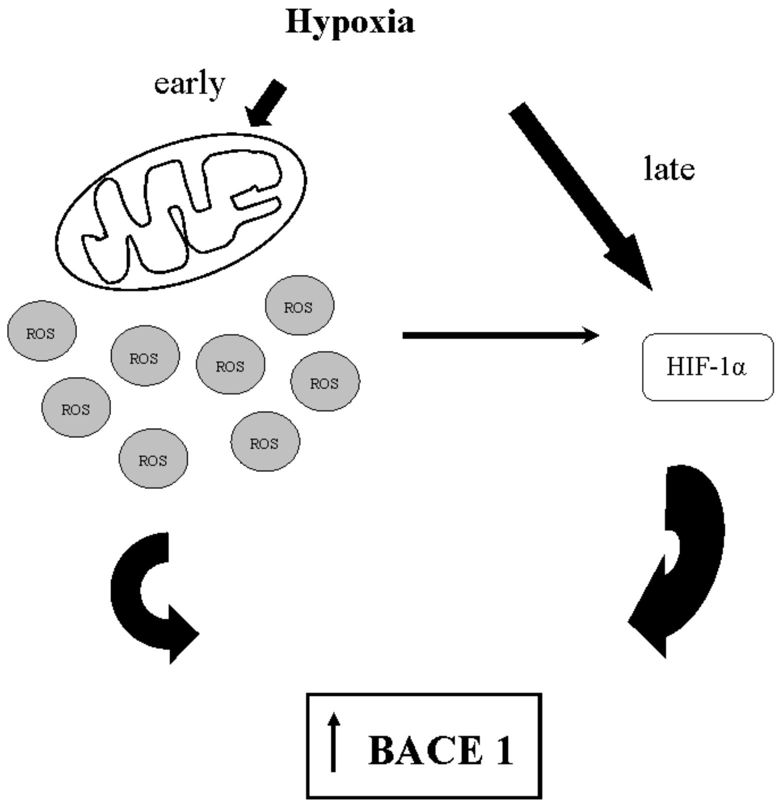

**FIGURE 3**. The early posthypoxic up-regulation of BACE1 depends on the generation of ROS mediated by the sudden interruption of the mitochondrial electron transport chain, while the later expression of BACE1 is caused by HIF-1α activation.

In addition, the OS-mediated up-regulation of BACE1 is mediated by the c-jun N-terminal kinase (JNK) pathway, as demonstrated by the silencing of JNK isoforms 1 and 2.

Instead, this pathway is not active in the late phase of posthypoxic BACE1 increase, which depends on the activity of HIF-1α, as shown by the data obtained by HIF-1α silencing.

It is well known that members of stress-activated protein kinase (SAPK), such as JNK, are markedly up-regulated in AD[87,88,89] and are activated by a variety of stress signals, including OS[90,91,92].

The early posthypoxic up-regulation of BACE1 recapitulates the cascade of events induced by oxidant agents and HNE in cells and in animal models. We have proposed that a sequence of events link ROS production, BACE1 up-regulation, and apoptotic cell death through an overproduction of Aβ. Initially, we have shown that oxidant agents and HNE significantly increase the expression, protein levels, and activity of BACE1 in $NT_2$ neurons, without affecting the levels of APP[31,32]. These events are followed by an overproduction of Aβ peptides as well as by morphological signs of apoptotic cell death[34]. Finally, it has been observed that the up-regulation of BACE1 is modulated by the JNK pathway. Thus, there is a significant correlation of BACE1 activity with oxidative markers in sporadic AD brain tissue[35], in which a significant increase of BACE1 expression has been shown[36,37,38].

Moreover, we and others[39,93] have recently found that BACE1 expression is regulated by γ-secretase activity, providing evidence of a positive feedback loop between the γ- and the β-secretase cleavages on APP. Interestingly, the expression of BACE1 is decreased by the activation of ERK1/2[94], which inhibits the γ-secretase[95].

The activity of the γ-secretase is modified by PS1 mutations as well as by molecules that interact directly with PS1[96,97,98]. OS is the only known factor able to augment the γ-secretase cleavage, increasing the expression of PS1, the catalytic subunit of the endoprotease.

These findings suggest that the overproduction of Aβ, dependent on the up-regulation of BACE1 as well as of the γ-secretase as induced by OS, contributes to the pathogenesis of the common sporadic, late-onset form of AD (Fig. 4).

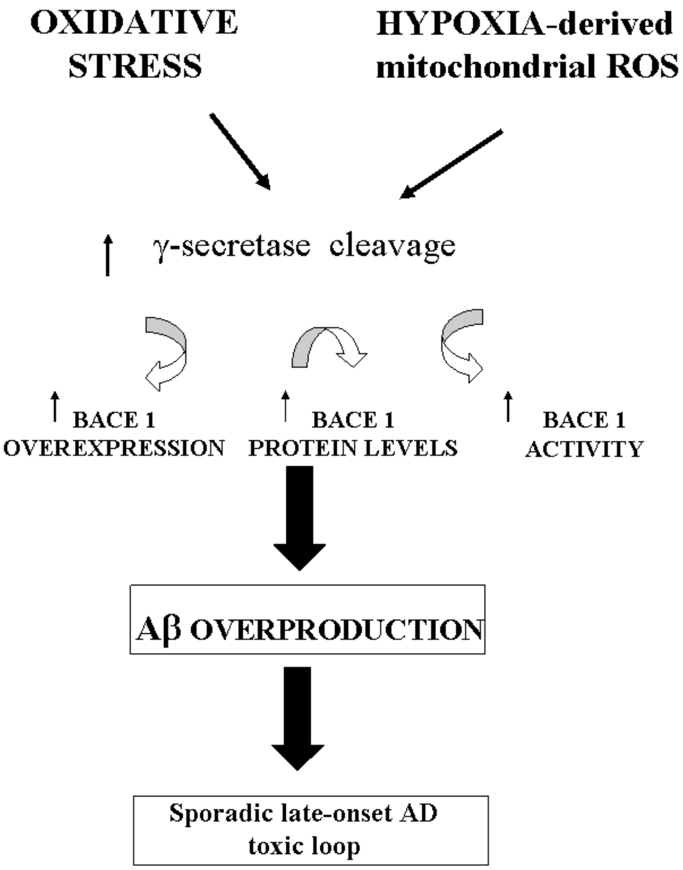

**FIGURE 4**. A sequence of the pathogenetic steps linking OS and/or hypoxia-mediated mitochondrial ROS, γ-secretase up-regulation, BACE1 induction, and overproduction of Aβ.

## ACKNOWLEDGMENTS

This study was supported by the Italian Ministry of Health (MT), CARIGE Foundation (MT), and Regione Piemonte (ET).
